# Targeting AMPK Networks for Male Reproductive Health: Mechanisms and Emerging Therapies

**DOI:** 10.3390/cells15090808

**Published:** 2026-04-29

**Authors:** Md Ataur Rahman, Abdel Halim Harrath, Maroua Jalouli, Jinwon Choi, Min Choi, Sohyun Park, Hyo Jeong Kim, Amama Rani, Salima Akter, Moon Nyeo Park, Bonglee Kim

**Affiliations:** 1 Department of Oncology, Karmanos Cancer Institute, Wayne State University, Detroit, MI 48201, USA; 2 Zoology Department, College of Science, King Saud University, Riyadh 11451, Saudi Arabia; hharrath@ksu.edu.sa; 3 Department of Biology, College of Science, Imam Mohammad Ibn Saud Islamic University (IMSIU), Riyadh 11623, Saudi Arabia; 4 Department of Pathology, College of Korean Medicine, Kyung Hee University, Seoul 02447, Republic of Koreashpark0912@khu.ac.kr (S.P.); amama.rani@khu.ac.kr (A.R.); mnpark@khu.ac.kr (M.N.P.); 5 Korean Medicine-Based Drug Repositioning Cancer Research Center, College of Korean Medicine, Kyung Hee University, Seoul 02447, Republic of Korea

**Keywords:** AMPK signaling, male infertility, metabolic infertility, testis, sperm mitochondrial function, metabolic regulation

## Abstract

Male infertility is an escalating global health issue, frequently associated with metabolic problems like obesity, diabetes, and age. Recent evidence designates AMP-activated protein kinase (AMPK) as a pivotal regulator linking energy balance to male reproductive function. AMPK regulates essential activities such as spermatogenesis, metabolic support of Sertoli cells, and steroidogenesis in Leydig cells, as well as sperm motility, capacitation, and the acrosome reaction. At the molecular level, AMPK coordinates signaling networks that include mTOR, SIRT1, PGC-1α, and FOXO to modulate mitochondrial function, oxidative stress, and autophagy-related quality control. Dysregulation of AMPK during metabolic and environmental stress results in compromised spermatogenesis, diminished sperm quality, mitochondrial malfunction, and reduced testosterone synthesis. Targeting AMPK signaling constitutes a possible therapeutic approach for enhancing male reproductive health. Pharmacological agents like metformin and AICAR, together with natural bioactive substances, lifestyle modifications, and exercise mimetics, have shown promise in reestablishing metabolic equilibrium and improving reproductive results. Moreover, combinatorial strategies that integrate antioxidants and autophagy modulators may yield synergistic advantages. Nonetheless, obstacles concerning tissue selectivity, optimum dose, and clinical translation persist. Future perspectives highlight precision medicine, biomarker-directed therapies, and the incorporation of metabolic health into fertility treatment. AMPK-targeted treatments collectively provide a novel and mechanistically sound method for addressing male infertility.

## 1. Introduction

Male infertility affects up to 40–50% of all couples who suffer from infertility worldwide [1]. In addition, over the past several decades, its incidence has been gradually increasing because of environmental factors, unhealthy lifestyle, and epigenetic changes [2]. Metabolic disorders, including obesity, diabetes mellitus, and metabolic syndrome have become one of the most prevalent causes of disrupted male reproductive function [3]. Metabolic dysfunction, driven by insulin resistance, hormonal imbalance, and oxidative stress, disrupts testicular microenvironment homeostasis, ultimately impairing spermatogenesis, sperm quality, and endocrine function [4]. Therefore, understanding how energy metabolism controls male reproduction and searching for potential targeted therapies remain a clinical priority.

AMP-activated protein kinase (AMPK), a highly conserved serine/threonine kinase that serves as the master regulator of energy homeostasis and stress signaling, is activated in response to various energy stressors including increased AMP/ATP ratio, hypoxia, and oxidative stress [5]. Once activated, AMPK signaling induces metabolic switch by activating catabolic pathways to produce ATP and shutting down ATP-consuming anabolic processes [6]. In addition to well-established metabolic effects, AMPK controls many important cellular processes such as autophagy, mitochondrial biogenesis, maintenance of cellular redox status and inflammation [7]. Many of these processes seem essential for normal male reproductive function due to high energetic requirements and tightly regulated differentiation processes during spermatogenesis and sperm maturation.

Emerging evidence suggest that AMPK might have essential functions in all aspects of male reproductive physiology [8]. In the testes AMPK signaling has been shown to regulate Sertoli cells that provide nutrition and metabolic support to germ cells as well as Leydig cells responsible for testosterone production [9]. AMPK signaling pathways also regulate sperm motility and capacitation through mitochondria and ATP production [10]. Dysregulation of AMPK has been linked to decreased spermatogenesis rate, accumulation of oxidative damage and lower sperm quality in men with metabolic syndrome [4]. Therefore, this review explores an important role of AMPKs at the crossroad of metabolism and male reproduction it might provide a novel potential target for treatment of male infertility. Additionally, activation of AMPK by pharmacological agents like metformin, lifestyle changes and natural bioactive compounds might provide opportunity to restore metabolic balance and correct male reproductive function through targeting AMPK signaling pathways.

This review transcends mere descriptive accounts of AMPK in reproductive biology by highlighting the therapeutic targeting of AMPK signaling networks. It incorporates pharmaceutical, behavioral, and molecular approaches to regulate AMPK activity, establishing a paradigm that links cellular energy control with clinically pertinent therapies. This work uniquely integrates metabolism–reproduction crosstalk with translational techniques, emphasizing how metabolic diseases hinder fertility via AMPK-mediated pathways. This review establishes a clear differentiation between preclinical and clinical findings, so augmenting scientific rigor and preventing overextrapolation. It also emphasizes novel precision medicine strategies, including biomarker-guided AMPK targeting, wherein semen-derived and metabolic biomarkers may facilitate patient categorization and individualized treatment [11]. This mechanism-to-therapy viewpoint differentiates the current study from previous studies and enhances its translational significance in promoting male reproductive health.

Existing reviews, such as Yang et al. (2020), primarily emphasize descriptive and mechanistic aspects of AMP-activated protein kinase (AMPK) in reproductive biology, despite the extensive research on this topic [8]. The focus is limited on therapeutic translation. There are significant voids in the integration of metabolism–reproduction crosstalk, the differentiation of preclinical from clinical evidence, and the establishment of actionable treatment strategies. This review addresses these limitations by incorporating precision medicine approaches, such as biomarker-guided therapy, and highlighting AMPK-targeted interventions within a mechanism-to-therapy framework. Furthermore, it investigates strategies for tissue-specific targeting to enhance clinical applicability. Therefore, this review explores to facilitate the advancement of AMPK-based therapies for male infertility by integrating fundamental biology with translational applications.

## 2. Semi-Systematic Literature Search Strategy

Databases searched: PubMed, Scopus, and Web of Science. Keywords used: combinations of “AMPK,” “male infertility,” “spermatogenesis,” “sperm motility,” “steroidogenesis,” “metabolic disorders,” and “AMPK activators”. Inclusion criteria: peer-reviewed articles in English focusing on AMPK signaling in male reproductive biology, including both mechanistic (in vitro and animal) and clinical studies. Exclusion criteria: non-peer-reviewed sources, unrelated systems, and studies lacking relevance to reproductive outcomes. Time frame: emphasis on recent studies while incorporating seminal earlier work where necessary.

## 3. AMPK Signaling Architecture in Male Reproductive Tissues

AMP-activated protein kinase (AMPK) is a heterotrimeric complex consisting of catalytic α (α1, α2), scaffolding β (β1, β2), and regulatory γ (γ1, γ2, γ3) subunits, which are encoded by separate genes and demonstrate tissue-specific expression patterns [12]. In the male reproductive system, the distribution of isoforms is significantly dependent on cell type, facilitating functional specialization within the testes and sperm. The α1 isoform is widely expressed in Sertoli cells, Leydig cells, and germ cells, where it modulates metabolic processes and stress responses [9]. Conversely, the α2 isoform is predominantly linked to metabolically active cells, such as developing germ cells and mature spermatozoa, and is essential for mitochondrial control and energy production [13]. The β subunits (β1 and β2) serve as scaffolds and possess glycogen-binding regions that affect subcellular distribution and enzymatic activity [14]. Recent research indicates that β2 is abundant in tissues with elevated metabolic requirements, such as testicular cells, which facilitate energy-demanding processes like spermatogenesis [4]. The γ subunits (γ1-γ3) function as energy sensors by binding AMP, ADP, and ATP, therefore regulating AMPK activity according to cellular energy levels [15]. The mix of isoforms functionally dictates AMPK sensitivity, location, and downstream signaling. This diversity facilitates the precise regulation of spermatogenesis, sperm motility, and steroidogenesis, underscoring the significance of isoform-specific targeting in formulating effective therapeutic options for male infertility.

AMPK is activated by an increased AMP/ATP or ADP/ATP ratio because of cellular stress that depletes cellular energy levels [16]. AMPK can also be directly phosphorylated and activated by upstream kinases such as liver kinase B1 (LKB1), Ca^2+^/calmodulin-dependent protein kinase kinase β (CaMKKβ) and transforming growth factor-β-activated kinase 1 (TAK1) [17]. Once AMPK is activated by phosphorylation of the α subunit at Thr172, AMPK acts to re-establish basal energy levels by activating catabolic pathways and inhibiting anabolic pathways [7].

**Figure 1:**
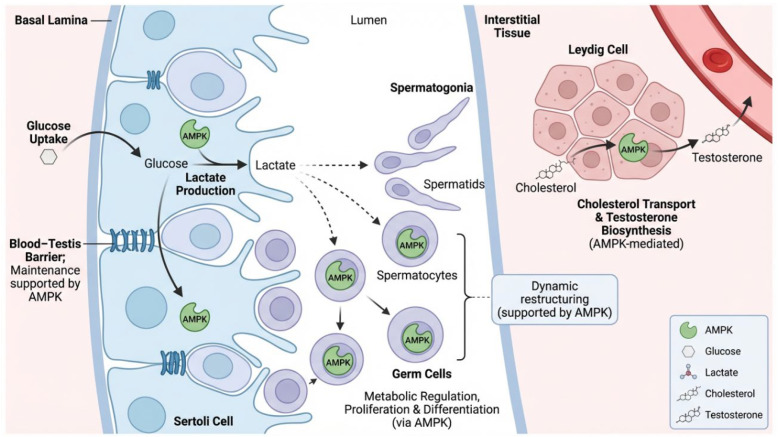
AMPK-mediated metabolic and functional regulation in male reproductive tissues. In the seminiferous tubules, AMPK in Sertoli cells facilitates glucose absorption and stimulates lactate synthesis, which acts as an essential energy source for maturing germ cells, such as spermatogonia, spermatocytes, and spermatids. AMPK has a role in preserving the blood–testis barrier and facilitates the dynamic cellular reorganization necessary for spermatogenesis. In germ cells, AMPK governs metabolic equilibrium, proliferation, and differentiation. In the interstitial compartment, AMPK in Leydig cells regulates cholesterol transport and testosterone synthesis, hence preserving endocrine function. AMPK collectively integrates energy metabolism, structural support, and hormonal regulation to maintain optimal spermatogenic development and male reproductive health.

AMPK exhibits tissue-specific patterns of regulation in male reproductive tissues for maintenance of testicular function [18]. AMPK is expressed in germ cells, Sertoli cells and Leydig cells within the testes [19]. It helps to regulate metabolism of these cells as well as proliferation and differentiation. In Sertoli cells, AMPK helps to regulate glucose uptake and lactate production ensuring ample metabolic support for developing germ cells [4]. AMPKα protein influences steroidogenesis in Leydig cells by regulating cholesterol trafficking and testosterone production [20]. There is also evidence to suggest that AMPK plays a role in maintaining the blood–testis barrier and supporting dynamic remodeling required for spermatogenesis [21]. The involvement of AMP-activated protein kinase (AMPK) in the regulation of metabolic and functional activities within male reproductive organs is depicted in Figure 1.

AMPK signaling pathway interacts with various other metabolic and stress responsive pathways [22]. AMPK signaling pathway interacts with various other metabolic and stress responsive pathways. AMPK directly inhibits mechanistic target of rapamycin (mTOR) signaling allowing for balanced cellular growth and autophagy [23]. AMPK is required for normal germ cell development and prevents germ cell tumors by inhibiting mTOR activity [19]. AMPK phosphorylates sirtuin 1 (SIRT1) which activates mitochondrial biogenesis mediated by peroxisome proliferator-activated receptor gamma coactivator 1-alpha (PGC-1α) [24]. AMPK signaling also modulates forkhead box O (FOXO) transcription factors, which result in the expression of genes involved in antioxidant responses, apoptosis, and longevity [25].

## 4. AMPK in Spermatogenesis and Testicular Cell Function

**Figure 2:**
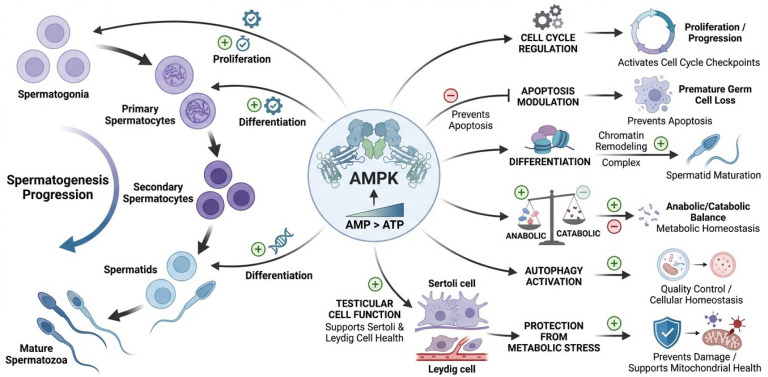
Regulatory role of AMPK in spermatogenesis and testicular cell function. When energy stress increases the AMP/ATP ratio, AMPK coordinates various germ cell development pathways. Spermatogenesis involves AMPK-induced germ cell proliferation and differentiation from spermatogonia to primary and secondary spermatocytes, spermatids, and mature spermatozoa. AMPK activates checkpoint pathways and controls apoptosis to prevent germ cell death. It enhances spermatid maturation by altering chromatin. AMPK also maintains metabolic stability in the testicular microenvironment by balancing anabolic and catabolic processes. AMPK activates autophagy to maintain cell quality and remove damaged organelles. Additionally, AMPK supports Sertoli and Leydig cell activity, boosting metabolic support and steroidogenic capacity while guarding against metabolic stress and mitochondrial malfunction.

By integrating cellular energy state with developmental and metabolic signals, AMPK plays a crucial role in coordinating spermatogenesis and maintaining testicular cell function [26]. This is accomplished through integrated cellular energy status. Spermatogenesis is a process that involves the proliferation and differentiation of germ cells from spermatogonia to mature spermatozoa [27]. This process requires a significant amount of energy and is strictly regulated through numerous steps. AMPK is responsible for regulating the destiny of germ cells by altering critical pathways that are involved in the progression of the cell cycle, apoptosis, and differentiation [28]. AMPK can maintain a healthy balance between anabolic and catabolic processes, which allows it to guarantee the development of germ cells in the correct manner while also preventing damage caused by metabolic stress [6]. In addition, the activation of autophagy through AMPK activity contributes to the maintenance of cellular quality control throughout the formation of germ cells. Figure 2 represents AMPK signaling in the regulation of spermatogenesis and testicular cell function. To maintain normal spermatogenesis and male reproductive health, AMPK is responsible for integrating energy sensing with cellular differentiation, survival, and homeostasis.

Like pancreatic β-cells, “nurse cells” called Sertoli cells provide metabolic and structural support to developing germ cells [4]. AMPK activation directly regulates glucose uptake and glycolysis in Sertoli cells, resulting in lactate production [29]. Since germ cells are unable to metabolize glucose, lactate becomes their primary energy source and depends on metabolic byproducts provided by Sertoli cells to promote germ cell survival and differentiation [4]. Additionally, AMPK has been shown to play an important role in maintaining the blood–testis barrier [8]. The blood–testis barrier consists of tight junctions between Sertoli cells that create a protective environment for developing germ cells by preventing toxic substances and blood-borne autoimmune reactions [30]. In addition to regulating tight junction proteins critical for this barrier, AMPK also regulates action and tubulin expression during spermatogenesis to allow for continuous remodeling.

AMPK signaling also plays an important role in Leydig cells, which are in the interstitial compartment of the testes and function primarily in steroidogenesis [20]. Leydig cells are responsible for testosterone production, which regulates spermatogenesis. AMPK appears to regulate cholesterol transport, which is the rate-limiting step in steroidogenesis, through regulation of steroidogenic enzymes [31]. While AMPK activation may acutely inhibit steroidogenesis during stress responses, basal activation of AMPK can also prevent excessive reactive oxygen species formation in Leydig cells [32]. Testicular AMPK dysregulation may lead to disrupted steroidogenesis and male infertility [4].

## 5. AMPK Activation and Control of Sperm Function

**Figure 3:**
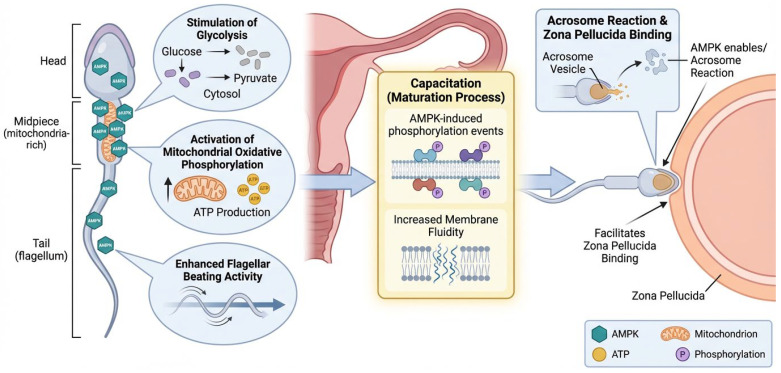
AMPK-mediated regulation of sperm energy metabolism, motility, capacitation, and acrosome reaction during fertilization. AMPK is situated in the sperm head, the mitochondria-dense midpiece, and the flagellum, where it regulates energy metabolism and movement. The activation of AMPK increases glycolysis in the cytosol, facilitating the conversion of glucose to pyruvate, and boosts mitochondrial oxidative phosphorylation, leading to higher ATP generation. This energy source facilitates flagellar movement and sperm motility. During capacitation, AMPK promotes phosphorylation processes and enhances membrane fluidity, aiding sperm maturation in the female reproductive tract. Moreover, AMPK governs the acrosome reaction by facilitating acrosomal vesicle exocytosis and augmenting sperm adhesion to the zona pellucida of the egg.

AMPK also affects sperm motility, capacitation, and acrosome reaction signaling during fertilization through energy metabolism [33]. To maintain their mobility for an extended period, spermatozoa cells need regulated energy input into the cell. AMPK in sperm cells acts as a metabolic sensor that ensures there is enough ATP produced and energy substrates used effectively [34]. AMPK helps control sperm motility by stimulating flagellar beating activity, glycolysis, and mitochondrial oxidative phosphorylation [26]. AMPK is also involved in sperm capacitation which matures the sperm once it reaches the female reproductive tract by initiating phosphorylation events necessary for fertilization and membrane fluidity [8]. Capacitation allows spermatozoa cells to undergo the acrosome reaction, where AMPK enables the sperm to bind to the zona pellucida of an oocyte [35]. Figure 3 depicts AMPK in modulating essential functional processes of sperm during fertilization. AMPK collectively integrates metabolic signaling with the anatomical and physiological changes necessary for successful fertilization, underscoring its critical involvement in male reproductive competence.

Mitochondrial function also plays an important role in sperm cells for proper bioenergetics. AMPK affects sperm function by initiating mitochondrial biogenesis and regulating oxidative phosphorylation necessary for ATP synthesis [36]. AMPK also helps maintain redox homeostasis in sperm cells by decreasing excess ROS production [37]. Physiological levels of ROS are important for signal transduction pathways during capacitation; however, oxidative stress can negatively affect spermatozoa cells leading to DNA fragmentation, lipid peroxidation, and apoptosis, damaging overall sperm function and infertility [38]. Treatment with AICAR activated AMPK and improved antioxidant activity in sperm cells decreasing oxidative damage under metabolic stress.

AMPK and autophagy/mitophagy also play roles in spermatozoa cells for quality control. Autophagy/mitophagy helps remove old or damaged organelles and defective sperm cells [39]. Activating AMPK can initiate autophagy pathways like LC3 which help regulate sperm cell quality [40]. Dysfunctional sperm mitochondria can occur if AMPK signaling is downregulated and inhibited.

## 6. AMPK Dysregulation in Male Reproductive Disorders

**Figure 4:**
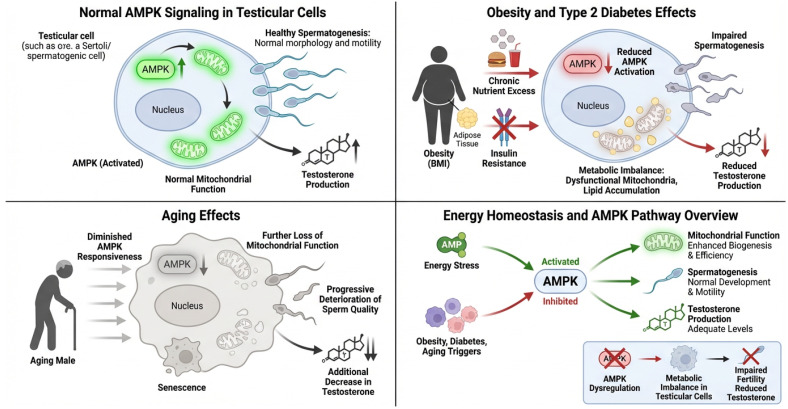
Dysregulation of AMPK on male reproductive function under metabolic and environmental stress. Normal spermatogenesis, sperm morphology, and motility are supported by AMPK activation in testicular cells, which increases mitochondrial activity, energy metabolism, and testosterone synthesis. Due to chronic food excess and insulin resistance, metabolic illnesses like obesity and type 2 diabetes limit AMPK activation, causing mitochondrial dysfunction, lipid buildup, poor spermatogenesis, and decreased testosterone synthesis. AMPK responsiveness decreases with age, causing cellular senescence, mitochondrial decline, sperm quality loss, and hormonal imbalance. Environmental and metabolic stresses alter AMPK-mediated energy homeostasis, causing testicular cell metabolic imbalance. The overview panel shows that AMPK dysregulation reduces testosterone and fertility, while correct activation boosts mitochondrial biogenesis, spermatogenesis, and endocrine function.

Emerging evidence suggests that the dysregulation of AMPK signaling contributes to male infertility. Metabolic disorders such as obesity, diabetes, and aging have been closely linked to infertility, conditions that are known to alter AMPK signaling and cause disturbances in energy homeostasis [41]. In obesity and type 2 diabetes mellitus, for example, excess nutrients and insulin resistance lead to decreased AMPK activation, contributing to an imbalance in cellular metabolism within the testes [3]. This dysregulation can lead to impaired spermatogenesis, decreased sperm motility, and reduced testosterone production [42]. In aged males, AMPK has been shown to become less responsive with time, which is associated with decreases in mitochondrial biogenesis, increased cell senescence, and a progressive loss of sperm function (Figure 4).

AMPK dysregulation also leads to increased oxidative stress and chronic inflammation in a male reproduction [43]. Decreased AMPK activity can cause impairment of the antioxidant system, leading to overproduction of ROS that can cause cellular damage to lipids, proteins, and DNA of germ cells and sperm [44]. Excess ROS can also disrupt mitochondrial function and promote inflammation, resulting in poor sperm motility and viability [44]. Additionally, inflammation-mediated signaling can be upregulated due to AMPK deficiency, leading to disturbances in the testicular environment and germ cell apoptosis [45]. Mitochondrial dysfunction and inflammation are common in many metabolic diseases and have been associated with male infertility [46]. Mitochondria are responsible for proper sperm motility and viability, and their dysfunction is closely tied to decreases in AMPK activity.

Environmental and lifestyle factors have also been shown to impact AMPK activity and cause disturbances in spermatogenesis and hormonal regulation [47]. Environmental toxins, endocrine-disrupting chemicals, heavy metals, and pesticides can all affect AMPK-mediated signaling, causing an increase in oxidative stress and disease [48]. Poor diet and exercise, smoking, and stress can all suppress AMPK activity and lead to metabolic dysregulation [49]. In summary, AMPK plays a critical role in the maintenance of metabolic regulation and fertility [50]. Disease states or environmental/lifestyle exposures that decrease AMPK activity can cause many of the same downstream effects that are commonly seen in men with infertility [51]. Understanding the mechanisms by which AMPK signaling affects sperm function and fertility can allow for therapeutic targeting of this pathway.

Environmental pollutants, such as heavy metals (e.g., cadmium, lead, mercury) and endocrine-disrupting chemicals (EDCs) like bisphenol A and phthalates, compromise male reproductive function by interfering with AMPK signaling pathways [52]. A key mechanism entails mitochondrial malfunction, wherein toxins disrupt electron transport chain activity, resulting in diminished ATP generation and modified AMP/ATP ratios [53]. While AMPK is often activated during energy stress, chronic mitochondrial damage can lead to dysregulated or inadequate AMPK activation, hindering cellular adaptability [54]. Moreover, these toxins stimulate the overproduction of reactive oxygen species (ROS), surpassing antioxidant defenses and resulting in oxidative damage to lipids, proteins, and DNA in testicular cells [45]. Increased ROS levels can interfere with AMPK signaling by altering upstream kinases, including liver kinase B1 (LKB1) and Ca^2+^/calmodulin-dependent protein kinase kinase β (CaMKKβ), thereby diminishing AMPK phosphorylation and activation [5]. Environmental contaminants trigger inflammatory signaling pathways, such as NF-κB, which can inhibit AMPK function and induce cellular stress responses [55]. Moreover, toxin-induced inhibition of AMPK results in the hyperactivation of mTOR signaling, suppression of autophagy, and accumulation of damaged organelles, ultimately compromising spermatogenesis and sperm functionality [56]. These pathways collectively demonstrate how environmental contaminants interfere with AMPK-mediated metabolic regulation, leading to oxidative stress, mitochondrial dysfunction, and male infertility.

## 7. Therapeutic Targeting AMPK for Male Reproductive Treatments

**Figure 5:**
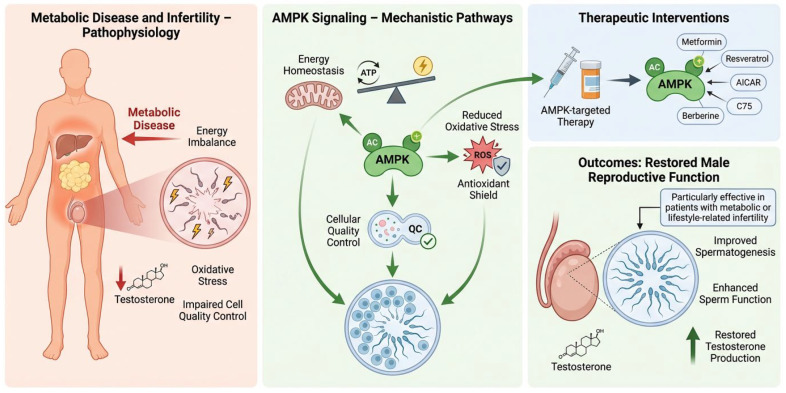
Therapeutic targeting of AMPK restores metabolic and reproductive functions in males. Metabolic diseases, including obesity and diabetes, cause energy imbalance, oxidative stress, impaired cellular quality control, and diminished testosterone synthesis, ultimately resulting in altered sperm function and infertility. The activation of AMPK reinstates energy balance by improving mitochondrial activity, diminishing reactive oxygen species (ROS), and facilitating cellular quality control processes, such as autophagy. Therapeutic approaches aimed at AMPK, including pharmaceutical drugs like metformin, AICAR, resveratrol, berberine, and C75, seek to reconfigure metabolic pathways and enhance testicular function. These therapies synergistically augment spermatogenesis, boost sperm quality and motility, and normalize testosterone levels.

Targeting AMPK signaling is a viable therapeutic strategy to restore metabolic equilibrium and enhance male reproductive function. AMPK-directed therapies can improve spermatogenesis, sperm function, and testosterone production by regulating energy balance, oxidative stress, and cellular quality control, especially in persons with metabolic diseases and lifestyle-related infertility [8]. Figure 5 depicts the involvement of AMPK in connecting metabolic dysregulation to male infertility and the therapeutic prospects of AMPK-targeted treatments.

### 7.1. Pharmacological AMPK Activators

The pharmacological stimulation of AMPK has become a promising therapeutic strategy for enhancing male reproductive function, particularly in relation to metabolic diseases [57]. Metformin, an extensively researched AMPK activator [58], primarily functions by inhibiting mitochondrial complex I, resulting in an elevated AMP/ATP ratio and consequent activation of AMPK [59]. In male reproductive systems, metformin has demonstrated the ability to enhance insulin sensitivity, diminish systemic inflammation, and mitigate oxidative stress, therefore fostering a conducive testicular milieu [60]. Experimental and clinical investigations indicate that metformin may augment spermatogenesis, enhance sperm count and motility, and partially reinstate testosterone levels in persons with obesity or type 2 diabetes [61].

AICAR (5-aminoimidazole-4-carboxamide ribonucleotide) is a well-defined AMPK activator that simulates AMP and directly stimulates AMPK activation [62]. AICAR has shown the capacity to improve mitochondrial activity, stimulate fatty acid oxidation, and modulate cellular energy equilibrium in preclinical models involving testicular cells [63]. These effects enhance sperm vitality and diminish germ cell death.

Additional pharmacological treatments, such as thiazolidinediones, salicylates, and novel small-molecule AMPK modulators, are under investigation for their reproductive advantages [64]. These substances may affect steroidogenesis, specifically by modulating cholesterol transport and essential steroidogenic enzymes in Leydig cells. Nonetheless, it is crucial to recognize that excessive or extended activation of AMPK may inhibit anabolic pathways, such as testosterone production, underscoring the necessity of dose optimization [65]. Pharmacological AMPK activators offer a mechanism-based strategy for restoring metabolic and reproductive functions; nevertheless, additional clinical validation is required to confirm long-term safety and efficacy in male fertility treatment.

### 7.2. Natural Bioactive Compounds and Lifestyle Interventions

Natural bioactive substances and lifestyle alterations provide as accessible and complimentary methods for activating AMPK and enhancing male reproductive health [66]. Numerous phytochemicals, including resveratrol, quercetin, curcumin, berberine, and epigallocatechin gallate (EGCG), have demonstrated the ability to activate AMPK signaling pathways and provide antioxidant, anti-inflammatory, and mitochondrial-protective benefits [67]. These chemicals augment cellular energy equilibrium by stimulating mitochondrial biogenesis, enhancing oxidative phosphorylation efficacy, and diminishing reactive oxygen species (ROS) production [68]. In the male reproductive system, these actions result in improved spermatogenesis, increased sperm motility, and safeguarding against DNA damage [69].

Resveratrol, a polyphenol included in grapes, activates AMPK via SIRT1-dependent pathways, enhancing mitochondrial activity and diminishing oxidative stress in testicular cells [70]. Curcumin and quercetin have been demonstrated to regulate AMPK and its downstream signaling pathways, such as mTOR and FOXO, therefore enhancing cellular resilience and spermatogenic efficiency [71]. Berberine, a phytogenic alkaloid, has exhibited analogous effects to metformin in stimulating AMPK and enhancing metabolic and reproductive metrics [72].

Lifestyle treatments significantly influence AMPK activity. Consistent physical exercise, calorie limitation, and balanced nutrition inherently stimulate AMPK by creating mild energy stress, thus improving metabolic flexibility and insulin sensitivity [34]. These therapies enhance hormonal equilibrium, decrease adiposity, and alleviate systemic inflammation, all of which contribute to improved male fertility results. Lifestyle-induced AMPK activation provides a sustainable and low-risk approach for maintaining long-term reproductive health [73]. Nonetheless, individual diversity in responsiveness to these therapies underscores the necessity for individualized strategies. The integration of natural substances with lifestyle adjustments may yield synergistic advantages, rendering this strategy especially beneficial in the management of metabolic and lifestyle-related infertility [74].

### 7.3. Exercise Mimetics and Metabolic Reprogramming

Exercise is a powerful physiological stimulator of AMPK, and exercise mimetics are intended to emulate these metabolic advantages for persons unable to participate in regular physical activity [75]. Compounds like AICAR and other novel drugs stimulate AMPK signaling pathways, facilitating mitochondrial biogenesis, augmenting oxidative metabolism, and optimizing energy usage [76]. These benefits are especially advantageous for sperm function, as sufficient ATP generation is crucial for motility, capacitation, and fertilization [36].

AMPK-mediated metabolic reprogramming transitions cellular processes from energy-intensive anabolic pathways to energy-generating catabolic pathways [34]. This alteration in testicular cells augments fatty acid oxidation, promotes glucose metabolism, and bolsters mitochondrial efficiency [77]. Consequently, sperm cells exhibit increased energy availability, less oxidative stress, and improved viability [78]. Exercise mimetics may enhance systemic metabolic health by diminishing insulin resistance and inflammation, hence indirectly supporting reproductive function [79]. This method is especially pertinent for individuals with obesity, inactive lifestyles, or metabolic syndrome. The long-term safety and efficacy of exercise mimetics, while intriguing, necessitate additional research [75]. Future research should concentrate on enhancing these drugs for specific AMPK activation in reproductive organs while reducing off-target effects.

### 7.4. Combination Strategies with Antioxidants and Autophagy Modulators

Combination techniques that incorporate AMPK activation alongside antioxidants and autophagy modulators provide a holistic treatment solution for male infertility [80]. Oxidative stress significantly contributes to sperm failure, resulting in lipid peroxidation, DNA damage, and compromised motility [81]. Antioxidants, including N-acetylcysteine, coenzyme Q10, vitamin C, and vitamin E, are essential for neutralizing reactive oxygen species and safeguarding sperm integrity [82]. Concurrently, AMPK activation facilitates autophagy, a cellular quality control process that eliminates damaged organelles and proteins [83]. This mechanism is crucial for preserving mitochondrial integrity in spermatozoa. Autophagy modulators can amplify this impact, facilitate the effective removal of defective mitochondria and diminish apoptotic signals [84].

The synergistic combination of AMPK activators and antioxidants simultaneously targets the root causes and downstream effects of metabolic dysregulation, enhancing therapeutic efficacy [85]. AMPK restores energy balance, antioxidants mitigate oxidative damage, and autophagy modulators promote cellular repair [34]. Integrated solutions are particularly advantageous in circumstances marked by chronic metabolic stress, such as obesity, diabetes, and aging [86]. Nonetheless, meticulous optimization of treatment combinations and dosages is essential to prevent possible harmful interactions. Combination therapy signifies a viable avenue for enhancing male reproductive outcomes.

### 7.5. Tissue-Specific Targeting Strategies for AMPK Activation in Male Reproductive Tissues

Developing tissue-specific activation of AMP-activated protein kinase (AMPK) in the testes is essential to optimize treatment effectiveness while reducing systemic metabolic disruption [87]. A promising strategy entails the utilization of tailored drug delivery systems, namely nanocarriers including liposomes, polymeric nanoparticles, and ligand-functionalized nanoconjugates [88]. These systems can be designed to preferentially concentrate in testicular tissue by integrating targeting ligands that identify receptors present on Sertoli or Leydig cells. Moreover, local testicular administration, encompassing intratesticular or peritesticular distribution, may augment drug concentration at the target region while minimizing systemic exposure; nonetheless, these methods necessitate thorough assessment for safety and viability [89]. Another technique entails the creation of isoform-selective AMPK agonists. The makeup of AMPK subunits differs among tissues, with specific isoforms, notably α2 and β2, being prominently expressed in metabolically active and reproductive organs [90]. Creating molecules that selectively activate these isoforms may enable more accurate control of AMPK signaling in the testes, thus minimizing off-target effects in other organs like the liver or muscle. Moreover, rational drug design concepts must prioritize the optimization of pharmacokinetics, tissue penetration, and receptor specificity to improve selective targeting. The conjugation of testis-specific peptides, utilization of biodegradable carriers, and implementation of controlled-release formulations can enhance localization and diminish systemic toxicity [91]. These efforts collectively establish a framework for the development of testis-targeted AMPK treatments, enhancing the translational potential of AMPK modulation in male reproductive medicine while resolving safety and specificity issues.

### 7.6. Population-Specific Considerations: Obese/Diabetic vs. Normometabolic Infertile Males

Clinical responses to AMPK-targeted treatments vary markedly between obese or diabetic guys and normometabolic infertile males, indicating divergent underlying pathophysiological processes [57]. In obese and insulin-resistant people, male infertility is frequently caused by metabolic dysfunction, encompassing hyperinsulinemia, chronic inflammation, oxidative stress, and disrupted hormonal control [92]. In these populations, AMPK activators like metformin typically exhibit more significant advantages, including enhancements in insulin sensitivity, testosterone levels, and, in certain instances, sperm characteristics [93]. Conversely, normometabolic infertile males may experience infertility attributable to genetic, idiopathic, or localized testicular causes instead of systemic metabolic dysfunction [94]. In these instances, the therapeutic effects of AMPK activation are less reliable and may predominantly affect cellular stress responses and mitochondrial function rather than systemic metabolic regulation [95]. The disparities highlight the significance of patient stratification, as AMPK-targeted treatments may yield greater efficacy in metabolically challenged people compared to normometabolic groups.

## 8. Human Clinical Evidence from Randomized Controlled Trials (RCTs) of AMPK Activators for Male Infertility

Although there is currently little human clinical data assessing AMPK activators in male infertility, material is rapidly becoming available, especially from randomized controlled trials (RCTs) utilizing the commonly used indirect AMPK activator metformin [19]. Men with metabolic diseases such obesity, insulin resistance, and type 2 diabetes—conditions that are closely linked to reduced reproductive function—have been the subjects of the majority of RCTs [96]. These studies show that metformin enhances systemic metabolic parameters that are important predictors of testicular function, including as glycemic management, insulin sensitivity, and inflammatory status [97]. Following metformin treatment, several clinical investigations have shown minor changes in reproductive hormone profiles, such as normalization of testosterone levels and a decrease in endocrine imbalance linked to hyperinsulinemia [98]. In humans, metformin is typically administered at clinical doses of 500–2000 mg/day, mostly for metabolic disorders [99]. Numerous studies show improvements in insulin sensitivity, hormonal balance, and, in certain situations, semen parameters in men with metabolic disorders within this range. Metformin has been demonstrated to increase spermatogenesis, decrease oxidative stress, and improve sperm motility in animal models, with effective doses usually falling between 50 and 300 mg/kg/day [100]. However, extended exposure or greater dosages (>300–500 mg/kg/day in rodents) may block Leydig cell steroidogenesis, which could lower testosterone levels because of excessive AMPK activation and anabolic pathway inhibition [101].

Improvements in semen indices, including sperm concentration, motility, and morphology, have also been noted in several RCTs, especially in subfertile men with metabolic syndrome [102]. These positive benefits are believed to result from AMPK-mediated improvements in energy metabolism, oxidative stress reduction, and mitochondrial function in testicular and sperm cells. It is crucial to remember that the clinical data is still inconsistent. Variability in findings is caused by variations in research design, patient groups, dosage, and treatment length, and not all trials indicate appreciable increases in reproductive outcomes. Furthermore, rather than measuring AMPK activity in reproductive organs directly, the majority of RCTs evaluate indirect outcomes including metabolic and hormonal alterations. AMPK activators, especially metformin, may provide therapeutic benefits in metabolically impaired men with infertility, according to current RCT-based data [101]. However, to determine long-term reproductive outcomes, appropriate treatment regimens, and conclusive efficacy, bigger, well-designed clinical trials are needed.

## 9. Clinical Association Studies Between Semen AMPK Activity, Metabolic Markers, and Infertility Prognosis

Although they are still in their infancy, clinical association studies examining the connection between male infertility prognosis, metabolic status, and semen AMPK activity offer significant translational insights. It is still technically difficult to assess AMPK activity directly in human semen or testicular tissue, and it is not frequently performed in clinical settings. However, research has investigated indirect indicators of AMPK-regulated pathways, such as metabolic parameters, oxidative stress, and mitochondrial function, which are strongly associated with sperm quality and reproductive outcomes [26]. According to clinical observations, males who suffer from metabolic illnesses such obesity, insulin resistance, and type 2 diabetes have poorer semen quality, which is marked by decreased sperm motility, concentration, and DNA fragmentation [103]. These situations are linked to increased oxidative stress and altered cellular energy balance, both of which are controlled by AMPK signaling. Reduced mitochondrial efficiency and increased amounts of reactive oxygen species in sperm cells from affected people indicate reduced activation of AMPK-related pathways [38]. Additionally, relationships between semen parameters and systemic metabolic markers, such as fasting glucose, insulin levels, lipid profiles, and inflammatory mediators, have been documented [104]. These correlations provide credence to the idea that metabolic dysregulation adversely affects reproductive potential through processes that may include compromised AMPK activation. Additionally, recent research has indicated that enhanced metabolic management is linked to better semen quality and reproductive outcomes, which suggests that AMPK activation may play a role [105]. Despite these results, a major drawback is the absence of standardized techniques for directly measuring AMPK activity in semen. To establish AMPK as a predictive marker and therapeutic target in male infertility, more clinical research utilizing molecular biomarkers and functional assays is required.

## 10. Preclinical and Clinical Evidence of AMPK Activity in Male Fertility

Both preclinical and clinical research provide evidence for the involvement of AMP-activated protein kinase (AMPK) in male fertility, albeit the strength of the evidence varies across these levels. Strong mechanistic support for AMPK as a crucial regulator of male reproductive function is provided by preclinical data from in vitro studies and animal models [106]. AMPK activation has been shown in mouse models to increase Sertoli cell metabolic support, accelerate spermatogenesis, and control Leydig cell steroidogenesis [107]. Furthermore, AMPK activation enhances sperm motility, viability, and DNA integrity by promoting mitochondrial activity, lowering oxidative stress, and stimulating autophagy [107]. In animal models, pharmacological stimulation of AMPK with drugs like metformin and AICAR has been demonstrated to reverse reduced fertility linked to metabolic dysfunction [108]. Human clinical data, on the other hand, is still more scarce and mostly indirect. Preclinical research on AICAR typically uses systemic dosages of 0.5 to 1.0 mg/g body weight (or 500–1000 mg/kg) [109]. At these levels, AICAR maintains sperm function, improves energy metabolism, and increases mitochondrial biogenesis [110]. On the other hand, prolonged or high-dose exposure has been linked to altered cellular proliferation, metabolic overstimulation, and possible inhibition of steroidogenic action. AMPK activity in semen or testicular tissue is not directly measured in human studies; instead, connections between metabolic health and reproductive outcomes are the main emphasis [111]. Improvements in metabolic indices and, in certain situations, semen quality and hormonal profiles are suggested by clinical trials utilizing AMPK activators, especially metformin [112]. The significance of AMPK-regulated pathways is further supported by observational studies that show relationships between metabolic indicators, oxidative stress levels, and the severity of infertility [113]. Crucially, a significant translational gap is highlighted by the discrepancy between preclinical and clinical research. Although preclinical research provides solid molecular insights, direct clinical confirmation is still lacking. To validate AMPK’s therapeutic potential for male infertility, future research should focus on creating trustworthy indicators of AMPK activity in human reproductive organs and carrying out well planned clinical studies.

## 11. Potential Adverse Effects of Excessive or Prolonged AMPK Activation

The male reproductive system may suffer from excessive or extended activation of AMP-activated protein kinase (AMPK), even if moderate activation is advantageous for preserving metabolic and reproductive equilibrium. Since AMPK blocks important anabolic pathways necessary for the transfer of cholesterol and the manufacture of testosterone, one of the main issues is the inhibition of steroidogenesis in Leydig cells [114]. Long-term AMPK activation can suppress steroidogenic enzymes, which may lead to lower levels of testosterone in the blood and compromised endocrine function [114]. Furthermore, persistent AMPK activation may upset the equilibrium between anabolic and catabolic processes, resulting in decreased cell proliferation and changed germ cell differentiation [115]. Spermatogenesis may be adversely affected by this imbalance, which could lead to a reduction in sperm production. While excessive autophagy is protective at healthy levels, prolonged activation of AMPK can also cause cellular stress or autophagy-associated cell death [116]. Furthermore, by encouraging ongoing energy stress, excessive AMPK activation may damage mitochondrial dynamics and perhaps lower the supply of ATP needed for sperm motility and viability [117]. Overactivation of AMPK has also been linked to unintentional metabolic inhibition and changed redox balance in some situations [118]. Crucially, these negative effects may be reversible once AMPK activity returns to normal and are often dose- and duration-dependent. Therefore, to guarantee treatment efficiency without impairing reproductive function, precise management of AMPK activity is crucial.

The “therapeutic window” for AMPK activation denotes the ideal range of activity that improves metabolic and reproductive functions while avoiding negative consequences [119]. Moderate AMPK activation enhances mitochondrial efficiency, diminishes oxidative stress, facilitates spermatogenesis, and sustains testosterone synthesis [120]. Excessive or sustained activation may inhibit anabolic pathways, especially steroidogenesis in Leydig cells, resulting in diminished testosterone levels [121]. Consequently, attaining a balanced and physiologically pertinent amount of AMPK activity is essential. This window is affected by dosage, treatment length, and individual metabolic conditions, highlighting the necessity for individualized and meticulously controlled therapeutic approaches to optimize efficacy while reducing hazards.

## 12. Challenges, Knowledge Gaps, and Safety Considerations

Despite significant progress, some important obstacles hinder the implementation of AMPK-targeted medicines in clinical practice for male reproductive health. A significant obstacle is the species-specific disparities between preclinical models and people. Rodent studies have yielded significant mechanistic insights into the regulation of spermatogenesis and steroidogenesis by AMPK; nevertheless, disparities in testicular physiology, spermatogenic cycles, endocrine regulation, and metabolic responses hinder direct extrapolation [122]. These differences highlight the necessity for meticulously designed human trials to corroborate preclinical findings.

An additional significant difficulty is the dose-dependent paradox associated with AMPK activation. Moderate activation of AMPK promotes cellular energy equilibrium, diminishes oxidative stress, and facilitates spermatogenesis; however, excessive or sustained activation may obstruct anabolic pathways, especially Leydig cell steroidogenesis [123]. AMPK can inhibit cholesterol transport and downregulate essential steroidogenic enzymes, resulting in diminished testosterone synthesis [114]. This dual role requires meticulous optimization of dosage regimens to preserve therapeutic efficacy while safeguarding endocrine function.

Another drawback is the absence of validated AMPK-specific indicators in human reproductive settings. Presently, direct evaluation of AMPK activity in semen or testicular tissue is not generally practicable in clinical environments. Most studies depend on indirect markers, such as oxidative stress indicators or metabolic measures, which do not explicitly represent AMPK signaling [124]. The lack of dependable biomarkers obstructs patient classification, therapeutic response tracking, and precision treatment strategies.

Moreover, regulatory and safety factors pose considerable hurdles. Pharmacological AMPK activators, while promising, may have systemic effects due to the widespread expression of AMPK. Long-term safety data in reproductive populations are scarce, and any off-target consequences require thorough assessment. Regulatory approval necessitates rigorous clinical trials that validate efficacy, safety, and reproducibility. Confronting these issues is crucial for progressing AMPK-based treatments towards practical use in male infertility.

## 13. Contradictory Effects of AMPK Activation on Steroidogenesis

Although AMPK activation is typically linked to advantageous metabolic and cytoprotective outcomes in the testis, conflicting evidence has arisen concerning its function in steroidogenesis, especially under circumstances of high or extended activation. Numerous in vitro and animal investigations indicate that supraphysiological activation of AMPK can inhibit testosterone production in Leydig cells [125]. Mechanistically, the activation of AMPK has been demonstrated to suppress the production of steroidogenic acute regulatory (StAR) protein, thereby restricting cholesterol transport into mitochondria, which is the rate-limiting step in steroid hormone manufacturing [114]. Moreover, AMPK may inhibit crucial steroidogenic enzymes, such as CYP11A1 and 3β-hydroxysteroid dehydrogenase (3β-HSD), hence further diminishing androgen synthesis [126]. This inhibitory action is associated with AMPK-mediated reduction in mTOR signaling, which diminishes anabolic activities essential for steroidogenesis. These findings underscore a paradox that is dependent on dosage and duration: moderate AMPK activation promotes metabolic homeostasis and testicular function, while chronic or excessive activation redirects cellular signaling towards catabolic dominance, thereby undermining endocrine function. These data highlight the necessity of establishing a specific treatment window for AMPK regulation. From a translational standpoint, they highlight that unregulated pharmacological stimulation may adversely affect testosterone synthesis, especially in individuals devoid of metabolic abnormalities. Thus, meticulous optimization of dosage, duration, and patient selection is crucial for the safe application of AMPK-targeted treatments in male reproductive medicine.

## 14. Future Perspectives

Future investigations into AMPK-targeted therapeutics for male reproductive health should include tactics that improve specificity, clinical relevance, and translational efficacy. The advancement of isoform-selective AMPK modulators signifies a pivotal direction. Considering that AMPK subunits have tissue-specific expression, especially the α2 and β2 isoforms in metabolically active and reproductive tissues, the development of selective agonists should facilitate targeted activation in testicular cells while reducing off-target systemic effects [127]. Progress in structure-based medication design and tailored delivery systems will be crucial in attaining this objective.

Secondly, precision therapy informed by semen-derived biomarkers presents a possible method for personalizing treatment. Biomarkers indicative of AMPK activity, mitochondrial function, oxidative stress, and sperm quality may enhance patient classification, enable early diagnosis, and allow for real-time monitoring of therapy responses [128]. The incorporation of molecular profiling with traditional semen analysis could markedly enhance predictive precision and provide tailored AMPK-targeted treatments.

The incorporation of metabolic control into infertility diagnostic and therapy protocols is crucial. Given that metabolic dysfunction significantly contributes to male infertility, including regular metabolic screening—encompassing glucose tolerance, lipid profiles, and inflammatory markers—into fertility evaluations can enhance clinical outcomes [129]. Integrating lifestyle modifications, including diet and exercise, with AMPK-targeted pharmaceutical approaches may establish a holistic and enduring treatment framework. These targeted directives highlight the shift from mechanistic comprehension to precision-oriented clinical implementation in AMPK-driven reproductive care.

## 15. Conclusions

AMPK serves as a pivotal regulator of male reproductive function, harmonizing cellular energy status with essential biological processes such as spermatogenesis, sperm motility, mitochondrial bioenergetics, autophagy, and steroidogenesis. AMPK regulates testicular homeostasis and promotes normal fertility through its interaction with key signaling networks, including mTOR, SIRT1, and FOXO. Dysregulation of AMPK signaling significantly contributes to male infertility, especially in the context of metabolic stressors such as obesity, diabetes, age, and environmental factors. Impaired AMPK activity affects mitochondrial function, elevates oxidative stress, diminishes sperm quality, and decreases testosterone synthesis, underscoring its importance as a mechanistic connection between systemic metabolic health and reproductive dysfunction. Third, targeting AMPK constitutes a promising therapeutic approach, although it necessitates meticulous optimization to guarantee clinical efficacy and safety. Pharmacological activators, natural substances, and lifestyle modifications have shown promise in reestablishing metabolic equilibrium and enhancing reproductive results. Nonetheless, obstacles concerning tissue selectivity, dosage, and long-term safety persist. Future advancements will rely on the creation of isoform-selective AMPK modulators, the application of biomarker-guided precision medicines, and the incorporation of metabolic control into infertility treatment. These observations underscore the significance of AMPK as a mechanistic driver and therapeutic target, linking metabolic regulation to male reproductive health and enhancing translational prospects in reproductive medicine.

## Data Availability

No new data were created or analyzed in this study.
